# The Current State of Subjective Training Load Monitoring—a Practical Perspective and Call to Action

**DOI:** 10.1186/s40798-018-0172-x

**Published:** 2018-12-20

**Authors:** Joseph O. C. Coyne, G. Gregory Haff, Aaron J. Coutts, Robert U. Newton, Sophia Nimphius

**Affiliations:** 1 Centre for Exercise and Sports Science, School of Medical and Health Sciences, Edith Cowan University, Joondalup, Western Australia Australia; 2 Human Performance Research Centre, Faculty of Health, University of Technology Sydney, Moore Park, New South Wales Australia

## Abstract

This commentary delivers a practical perspective on the current state of subjective training load (TL) monitoring, and in particular sessional ratings of perceived exertion, for performance enhancement and injury prevention. Subjective measures may be able to reflect mental fatigue, effort, stress, and motivation. These factors appear to be important moderators of the relationship TL has with performance and injury, and they also seem to differ between open and closed skill sports. As such, mental factors may affect the interaction between TL, performance, and injury in different sports. Further, modeling these interactions may be limited due to the assumption that an independent signal can adequately account for the performance or injury outcomes. An independent signal model does not accurately reflect training environments where multiple stressors (e.g., mechanical, emotional, nutritional) impact adaptations. Common issues with using subjective TL monitoring, including a lack of differentiation between biomechanical, physiological, and cognitive load, may be overcome by considering psychometric measurement best practices, finer graded scales, and differential ratings of perceived exertion. Methods of calculating TL, including different acute and chronic time periods, may also need to be individualized to different sports and potentially different individuals within the same sport. As TL monitoring is predominately a “chronic” decision-making tool, “acute” decision-making tools, e.g., subjective wellness and autonomic nervous system measures, should be combined in a bespoke multivariate model to aid sports coaches. A call to action is presented for future research on key issues associated with TL monitoring that will have relevance for practitioners in an applied setting.

## Key points

- Subjective measures of training load may be able to reflect mental load, which appears to be an important moderator of training load’s relationship with performance and injury.
- The relationship between training load, performance, and injury may differ between open and closed skill sports due to mental load.
- Subjective measures of training load are recommended in bespoke multi-factorial models assessing the relationship between training load, injury, and performance.
- Future developments in training load monitoring should include quantifying the relationship between subjective measures, performance, and injury and establish preferred training load model calculations.

## Introduction

Training load monitoring is typically an attempt to quantify two interrelated relationships: the training load–performance relationship (TL-P) and training load–injury relationship (TL-I) [1]. Both of these relationships appear to be quadratic whereby if too much or too little training is completed, there is a higher likelihood of not performing well or becoming injured/ill [1, 2]. There are two general TL constructs: internal and external. These constructs, along with their interaction, have been described previously [1–4]. In practice, methods of monitoring TL vary considerably depending on the type of sport or activity [3]; however, TL models are commonly analyzed using training impulse, which is normally a product of an intensity factor and volume/duration factor [2, 5].

Subjective measures of TL, and in particular, sessional ratings of perceived exertion (sRPE), are recommended as a primary measure of TL in systematic reviews of the literature [6, 7]. Subjective measures may also be more sensitive and consistent than objective measures [8, 9], and sRPE has been reported as the most commonly assessed TL variable in most sports [10]. Besides sRPE, there are other subjective methods of assessing an athlete’s response to training, e.g., visual analogue scales [9] and perceived wellness/stress questionnaires [11]. Although these measures will be highlighted later in the commentary, they are not usually incorporated into common TL models using training impulse as intensity factors. Due to the above factors, sRPE will be the principal focus of this commentary.

A basic model of an athlete’s response to training can be estimated from data collected as part of the TL monitoring process [1, 5]. Specifically, the difference between “fitness” (positive) and “fatigue” (negative) functions can be quantified with internal or external TL variables as training impulse [1, 5]. The genesis of this basic TL model stems from the work of Bannister [5]. A recent simplified extension of this work has been the development of the acute to chronic workload ratio (ACWR) [12–14]. TL monitoring and in particular the ACWR has been readily adopted (especially in open skill sports) to inform training practices to minimize the likelihood of injury [1]. The ACWR has also been used as a tool to systematically progress injured athletes’ rehabilitation and to quantify acceptable levels of injury risk prior to an athlete returning to competition [14, 15]. The research on the ACWR suggests values above or below ~ 0.8–1.3 are associated with an increased risk of injury [12–14]. However, the level of evidence for this recommendation is not yet well developed and it is typically not advocated that practitioners completely avoid ranges outside ~ 0.8–1.3. These ranges may be practically impossible or undesired in certain situations like early rehabilitation and tapering. Practitioners should instead be cognizant that a higher injury risk may be present and combine this with other factors to make decisions.

Despite the association with injury risk, TL monitoring and ACWR currently appear to be poor predictors of injury [2, 16]. This poor predictive power in regard to future injury has led some practitioners to question the use of TL monitoring. However, it is important to understand that the ability of single variables to predict injuries will be limited considering the multitude of factors that may influence injury risk, including genetics [17], previous injury history [18], psycho-social stress [19–21], different psychological coping strategies [22], and even coaching style [23]. Additionally, the use of inconsistent injury reporting methods (e.g., “medical attention injuries” versus “match time loss only”) and the small number of injuries typically seen in many studies makes it difficult to compare results between studies [6, 24]. One solution to this issue is to adopt a universal injury categorization tool like the Subsequent Injury Classification Model 2.0 [25]. Similar to TL-I, there are limitations in the ability of single metrics to estimate performance with precision. Many factors including nutritional status [26], percentage of training affected by injury [27, 28], and coaching style [29, 30] affect training adaptations and subsequent performance. Notwithstanding these limitations, TL monitoring is still considered an important part of the athlete monitoring process [1, 3]. To improve this process, a multi-factorial approach that considers an athlete’s daily readiness to train and informal variables like sports coaches’ experience and understanding of athletes is recommended to enhance the understanding of TL-I and TL-P [31, 32].

In light of our improved understanding of the impact psychosocial/cognitive factors have on performance and injury, recent examinations of periodization theory and advances in using subjective TL measures, the purpose of this commentary is to provide a practical perspective on the current state of subjective training load (TL) monitoring for performance enhancement and injury prevention. It would also seem timely to reevaluate the importance and weighting of subjective TL measures. Common limitations associated with subjective TL monitoring will also be addressed as well as possible evidence-informed solutions to these issues. Further, a critical examination of common methods of calculation used in TL monitoring models is also warranted.

## The Impact of Psychosocial Factors on Injury and Performance

Despite appearing to be important moderators of TL-I and TL-P [19, 20, 33, 34], non-physical mental factors like mental fatigue, effort, stress, and motivation have received comparatively little attention to physical factors. Mental fatigue is a psychobiological state caused by demanding cognitive activity relative to the mental effort and motivation required to perform a task [34, 35]. Mental fatigue can be considered a component of mental stress, which may manifest in different forms including varied maladaptive coping behaviors (e.g., self-blame) and low perceived motivation [21, 36].

**Fig. 1:**
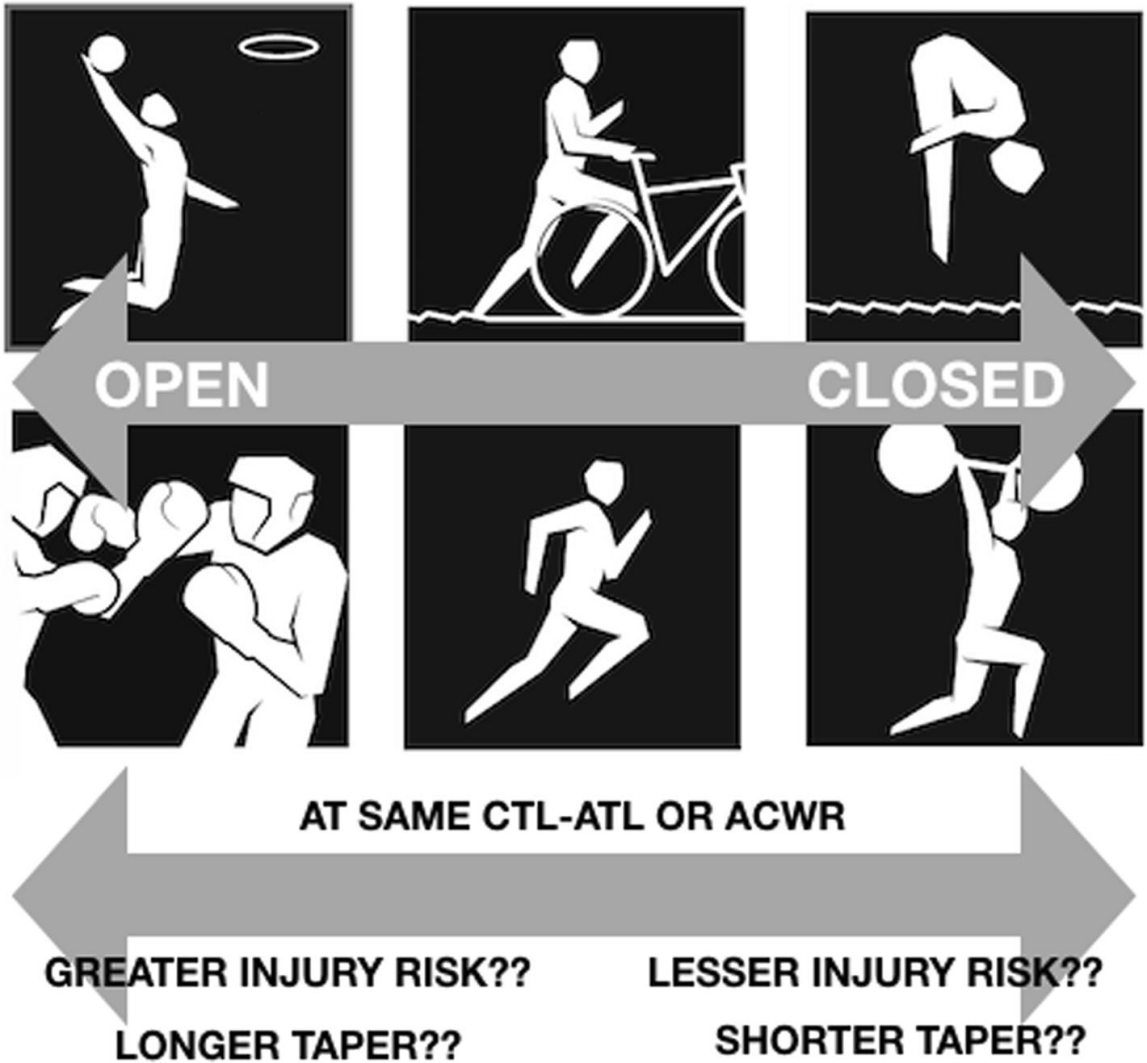
Possible interaction effects of open and closed skill sports on training load variables, performance, and injury risk. Individual London 2012 Olympic pictograms reproduced in complete figure with permission from the International Olympic Committee. CTL, chronic training load; ATL, acute training load; ACWR, acute to chronic workload ratio

It is notable that mental factors and mental load seem to differ between open and closed skill sports. Open skill sports (e.g., basketball, table tennis) require players to react in an unpredictable and changing externally paced environment while closed skill sports are performed in an environment that is relatively predictable, consistent, and internally paced (e.g., shot put, swimming) [37]. Due to these differences, open and closed skill sports place very different mental demands on athletes, and these demands develop different mental qualities [38–42]. For instance, athletes in open skill sports may develop better visual attention, action execution, and decision-making skills compared to closed skill sports athletes [40–42]. It is logical to suggest that the mental fatigue/stress from sports demands may moderate both TL-I and TL-P [21, 34]. Hypothetically, open skill sports may have greater incidences of injury at the same ACWRs and may possess a very different optimal TL-P relationship (i.e., require a longer taper to allow for extra mental load to dissipate) when compared to closed skill sports. This theorised relationship is detailed in Fig. 1.

## Updates to Periodization Theory

Coinciding with our increased knowledge of the impact non-physical factors have on performance and injury, it is interesting to note theories underpinning training periodization have recently been questioned [32, 43]. Modeling performance and injury is an associated extension of periodization theory [43]. A major assumption and limitation related to current TL models is that mechanical training stress or external TL is an independent signal for “fitness”/“fatigue” adaptations. However, an independent signal model does not accurately reflect training environments where multiple stressors (e.g., mechanical, emotional, nutritional) impact athletic adaptations and performance [26, 29, 33], nor does it reflect recent consensus definitions of TL [3], advances in the general adaptation syndrome model that consider non-mechanical training stress [43] and more contemporary allostatic and cognitive appraisal stress theories [21, 32, 44]. These contemporary stress theories suggest there is a complex and collaborative emotional, physiological, immunological, and psychological response driven by the brain’s perceptions of stressors [32, 44].

The allostatic stress and cognitive appraisal paradigms may aid our understanding in effectively evaluating TL measures. Subjective assessments like sRPE may reflect both the allostatic stress and the cognitive appraisal of the stress as it theoretically encompasses both the mechanical stress applied and also the (conscious) perception of that stress [32]. Compared to objective measures, sRPE may be able to better account for the allostatic stress athlete experience in mixed training sessions (e.g., tactical, skill, strength, fitness) [45, 46] and the mental load (e.g., learning a new skill or tactical strategy, competing against an unfamiliar opponent) during these sessions [47, 48]. However, it is currently unknown exactly how mental load affects sRPE, particularly in team-based open skill sports.

Although subjective measures may have advantages as stand-alone measures, practitioners should not discount established valid objective measures when monitoring TL. It is accepted that internal and external TL measures should be used or compared with multiple variables to better understand TL-I and TL-P [31]. It is also suggested that both subjective and objective internal TL measures should be viewed as interrelated but different constructs [31]. For instance, previous studies have reported ~ 50% unexplained variance between objective heart rate (HR)-derived measures and sRPE measures [31]. This highlights that both objective and subjective TL measures are not interchangeable and can give very different information. As such, if subjective measures provide a better illustration of “allostatic” load, the weighting of and comparisons between TL variables may need to be reconsidered for best practice. Currently, it is common in sports science to compare subjective perceptional measures (sRPE) to both objective internal (e.g., HR) and external (e.g., GPS-derived running measures) TL measures for validity purposes [45, 49]. However, a paradigm shift may be warranted whereby objective measures are compared to the subjective as the criterion to determine construct validity [50]. In regard to determining which TL measures to implement for practitioners, the authors suggest sRPE would seem to be a logical first choice due to the aforementioned reasons and low cost [1]. However, TL measures should be chosen on a case-by-case basis depending on the nature of the sport (e.g., professional road cycling may choose sRPE as a secondary measure behind power output and HR response) and characteristics of training environment (e.g., logistics, budget). It also bears repeating practitioners should not expect to be able to use one sole variable to fully explain internal or external TL, e.g., sRPE will not fully explain HR response to training.

It has been also suggested that a further step to qualify sRPE as a useful TL measure is to compare it against the changes in injury rates or performance to [31]. This practice may be misleading at present when considering the lack of consensus injury classifications [24, 25] and the difficulty sports science has in adequately defining performance (especially in open skill team sports) [51, 52]. Despite the inconsistencies in injury classification in prior research, subjective TL monitoring with sRPE seems associated with injury risk [16]. However, as mentioned prior, it is currently a poor predictor of future injury as a sole variable [16]. In regard to the difficulties in defining performance, the authors feel it is important to differentiate prior research examining subjective TL monitoring with performance in physical tests (e.g., a countermovement jump) compared to performance in actual sports competition as these may very well be unrelated [52].

When examining the performance outcomes in competition, there is very little research in this area, but there does appear to be some relationship with subjective TL monitoring and performance in both open [46, 53, 54] and closed skill [49, 55, 56] sports. However, as lower injury rates are significantly associated with competitive success in both open and closed skill sports [27, 57], this relationship with performance outcomes may simply be due to lower injury rates and not any boost in performance per se [27]. For instance, effectively applying subjective TL monitoring may lead to a reduced injury rate through better management of injury risk, which in turn would likely improve performance. Whether this distinction is meaningful to practitioners is another question altogether as increased performance is desired in most cases, regardless of means. However, at the moment, the level of evidence to support the effectiveness of using subjective measures in similar “fitness”/“fatigue” models like the ACWR to monitor and manipulate training for performance purposes is not yet well established [52].

## Subjective Training Load Monitoring: Limitations and Possible Solutions

**Fig. 2:**
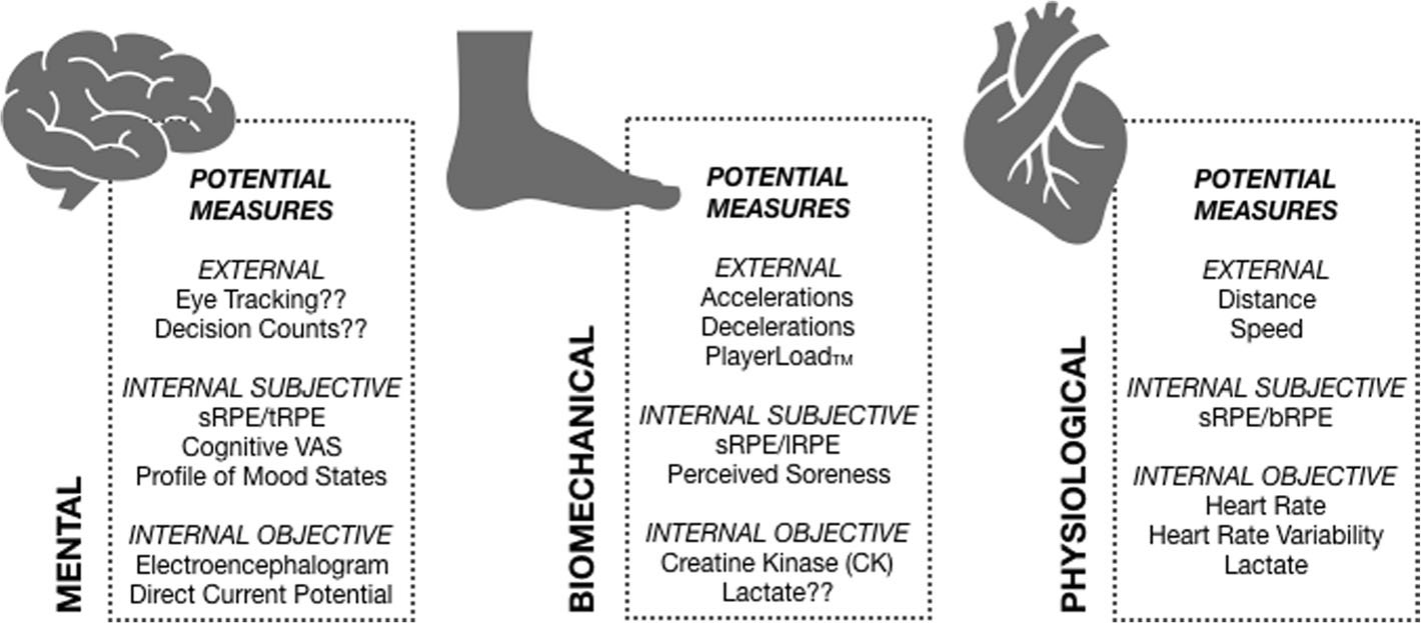
Potential measures to combine with differential RPE or sRPE in a multivariate training load model that considers physiological, biomechanical, and mental load. Adapted from Vanrenterghem et al. [63]. sRPE, sessional rating of perceived exertion; tRPE, technical rating of perceived exertion; lRPE, leg muscle rating of perceived exertion; bRPE, breathe rating of perceived exertion (breathlessness); VAS, visual analogue scale

Limitations of using sRPE to monitor TL and their possible solutions should also be acknowledged [58]. The first potential limitation of using sRPE relates to the purpose for using TL monitoring. Although sRPE may give a better representation of overall load on the athlete, it should not be interpreted as a representation of physiological or biomechanical load. If physiological or biomechanical load are of paramount importance to sports’ training practices (e.g., heart rate and power output in professional road cycling), sRPE may be of less relevance. Other limitations include practical errors common in recording sRPE. These include using non-validated sRPE scales (e.g., linear scales or scales without verbal anchors) and failing to obtain individual responses (e.g., peer presence on the rating of sRPE). Anecdotally, poor education of athletes as to the importance of giving accurate responses and how sRPE will be used by practitioners is commonly reported. If education around sRPE is not adequate, athletes may answer dishonestly in an attempt to manipulate future training sessions or team selection. Using a global sRPE score with the common 10-point category ratio scale (CR10) may also reduce the measure’s sensitivity to account for the range of biomechanical and physiological exertion demands across training [7] and fail to distinguish between psychophysiological responses to training stress [48]. To counter these issues, it is recommended that practitioners follow the fundamental rules of psychometrics in administering sRPE or any subjective measure [11]. To improve sensitivity, a 100-point RPE category ratio scale (CR100) should be considered due to more verbal anchors and a finer grading compared to the CR10 scale [59–61]. To examine different components of training stress, practitioners may also consider differential RPE. Differential RPE refines how athletes rate different components of training/performance and requires separate scores for combinations of breathlessness (bRPE), leg muscle exertion (lRPE), upper body exertion (uRPE), and technical/cognitive exertion (tRPE) and in some cases match exertion (mRPE) [47, 48, 62]. With these components, differential RPE seems to encompass perceptions of separate physiological (bRPE) and biomechanical load (lRPE) [63] while also accounting for mental load (tRPE). As an example, tRPE in isolation appears sensitive to the quality of opposition in English Premier League soccer [47]. Differential RPE may also enhance measurement precision and sensitivity and improve within-athlete reliability compared to traditional global sRPE scores [62, 64]. Practically, separate scores (e.g., bRPE, lRPE, tRPE) may be averaged to give a global RPE score if desired [47]. However, practitioners will need to weigh these potential benefits against the increased number of measures and reporting requirements with differential RPE. These increased requirements are a potential practical limitation and may affect athlete compliance. Figure 2 provides an example of how sRPE or differential RPE may be combined with other well-known measures to create a multivariate TL model that considers physiological, biomechanical, and mental load [63].

## Training Load Model Calculation Methods

There are also limitations in how various TL metrics, like the ACWR, are calculated. Specifically, there has been a debate over the arbitrary length of acute and chronic periods and deciding what training components should be included in the models [46, 65, 66]. Additionally, there has also been criticism over appropriate calculation methods. Rolling averages (which is the most common method used to calculate the 7- and 28-day periods of the ACWR) may not account for variations in the way TL is accumulated and the decaying nature of “fitness” and “fatigue” [1, 67, 68]. As such, it has been suggested that exponentially weighted moving averages (EWMA) may be a superior alternative [67–69]. However, there are also conceptual issues with EWMA and other TL models when considering athletes will likely have individual decay rates of both “fitness” and “fatigue.” For instance, a recent study on rugby sevens players used the Bannister impulse response model to predict heart rate variability (as a substitute for performance measures) from sRPE [70]. The mean “fitness” and “fatigue” decay rates for sRPE were 20 ± 14 and 11 ± 7 days respectively [70]. Despite the method of determining “fitness” and “fatigue” decay rates being questionable, the arbitrary 7- and 28-day acute and chronic period lengths commonly used to determine the ACWR may not be appropriate for all sports or individuals [2, 65]. Using different period lengths would seem relevant to athletes adapted to different micro- and mesocycle lengths (e.g., microcycles 3–10 days, mesocycles 2–6 weeks) or who are within uncommon training periods (e.g., competition periods or returning from injury). Examinations of different acute and chronic time frames have resulted in varied relationships with injury risk [2, 65]. For example, 3- or 6-day acute and 21-day chronic periods best explained injury risk in one investigation involving Australian football players [65]. However, when accounting for the interaction of absolute chronic TL levels with injury risk, another investigation in the same sport found no improved predictive capacity above the common 7- and 28-day periods compared with combinations in acute periods of 7 and 14 days and chronic periods of 21, 28, 35, 42, 49, and 56 days [2]. From this research, practitioners may be able to adjust the length of acute and chronic periods in the ACWR based on the lengths of their preferred training micro- and mesocycles [2]. However, as mentioned, it is also worth considering acute and chronic periods may be both individual and idiosyncratic to different sports and may require calibration to an objective measure [5]. Further consideration of which training components (e.g., technical, resistance, recovery) should be included in TL models for association or prediction purposes is also warranted. A recent study on Australian football players suggested a model using only skill training sRPE is better for predicting performance than a model using total sRPE [46]. Hence, a possible advancement may be to record separate technical and non-technical training sRPE scores and differentiate between each in analyses. However, the level of evidence for idiosyncratic acute and chronic periods and distinguishing between technical and non-technical training sRPE in TL models is not yet well developed.

## “Acute” and “Chronic” Decision-Making Tools

**Fig. 3:**
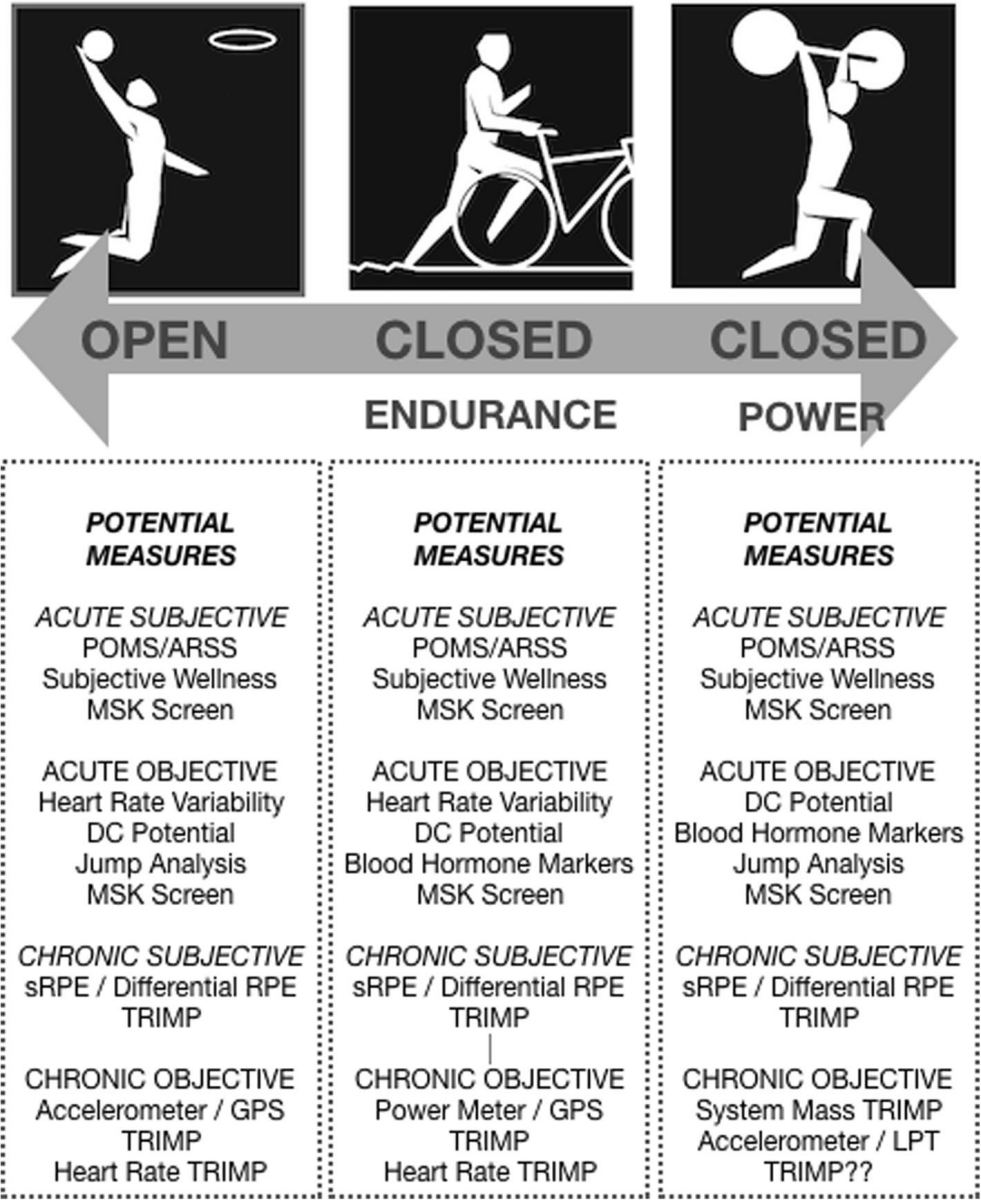
A potential framework of “acute” and “chronic” decision-making tools for different types of sports. POMS, Profile of Mood States; ARSS, Acute Recovery and Stress Scale; MSK, musculoskeletal; DC, direct current; LPT, linear position transducer; GPS, Global Positioning System; RPE, rate of perceived exertion; TRIMP, training impulse. Individual London 2012 Olympic pictograms reproduced in complete figure with permission from the International Olympic Committee

Additional limitations with TL monitoring using training impulse (e.g., the ACWR) is that they are predominately “chronic” decision-making tools (e.g., how to structure training from week to week). They also rely upon post-training analysis. It should be noted that the ACWR can be calculated and compared daily in an attempt to assess athlete adaptations, e.g., an internal TL increase at the same external TL may signify maladaptation. However, this practice may not be sensitive enough to inform daily training modifications [71] which becomes problematic when “acute” decisions, like modifying training based on day-to-day athlete readiness, are required. Although dependent on context, it is the authors’ experience that most high-level coaches (e.g., world record/multiple Olympic games and professional team sports coaches) prefer tools that aid in these “acute” decisions (i.e., “Do I need to make a change today? And if so, by how much?”). “Chronic” decision-making information may not be as highly valued by such coaches, and explanations of ACWR principles are typically well known when given in familiar language. This preference for “acute” tools may reflect an attempt by coaches not to become “trapped” in any non-opportunistic rigidity associated with long-term planning (a management theory principle) [72, 73], especially when athletes’ needs/priorities may change daily. Examples of “acute” tools may include both subjective (e.g., perceived wellness scales [11]) and objective (e.g., heart rate variability [74]). It would then seem important for practitioners to quantify which “acute”/“chronic” decision-making tools coaches feel can best aid their practice and implement them on a bespoke basis. The authors suggest athlete-monitoring tools can be divided into four categories: (i) “acute” subjective, (ii) “acute” objective, (iii) “chronic” subjective, and (iv) “chronic” objective. A list of possible “acute” and “chronic” decision-making tools for different types of sports (e.g., closed versus open) and how practitioners may combine them with sRPE/differential RPE are provided in Fig. 3 [3, 74]. Besides coach preference, the usefulness of these “acute” decision-making tools may vary from sport to sport. For instance, when compared to central nervous system measure like direct current potential, autonomic nervous system measures (e.g., heart rate variability) may not be very meaningful for explosive sports like weightlifting or track and field jumps and throws. Similar to TL measures, these measures should be recognized as providing different information, e.g., autonomic nervous system status may be practically unrelated to central nervous system status in an athlete. Further investigations of the utility of “acute” decision-making tools in different sports, their relationship with TL measures, and other informal variables like coach experience and intuition would seem warranted.

## Call to Action

Currently, the level of evidence supporting the efficacy of TL monitoring systems, and in particular, the ACWR, is not high. As such, there are a number of considerations presented in this article practitioners should take into account when implementing TL monitoring in their practice. Based on these considerations, the authors put forward a call to action for further examinations of the key issues identified in this commentary that would be of use in an applied setting. These issues include quantifying the ability to assess mental load with current subjective measures, the contribution of mental load to existing subjective measures and establishing if mental load differs between open and closed skill sports. From here, examinations into how the ability to handle mental load may moderate TL-I and TL-P relationships (similar to previous examinations on physical capabilities) would be of interest. The relationship between subjective measures, performance, and injury should also be further investigated in both open and closed skill sports. These further investigations should attempt to quantify competition performance outcomes independent of injury rates to determine if models like the ACWR are effective in modeling performance in of itself, i.e., distinct to benefits of improved training availability due to lower injury rate. They should also encompass which methods of TL model calculation (e.g., rolling averages and EWMA, different acute and chronic periods, varying “fitness”-“fatigue” decay rates) have stronger relationships with performance and injury. “Acute” decision-making tools should be also assessed against TL models and for their suitability in different sports under common practical conditions athletes face. Lastly, it seems important for practitioners to identify which methods of monitoring are most important to sports coaches in their practice and fit those into a bespoke multi-factorial model.

## Abbreviations

ACWR: Acute to chronic workload
bRPE: Ratings of breathlessness
CR10: 10-point category ratio scale
CR100: 100-point category ratio scale
EWMA: Exponentially weighted moving averages
HR: Heart rate
lRPE: Ratings of leg muscle exertion
mRPE: Ratings of match exertion
sRPE: Sessional ratings of perceived exertion
TL: Training load
TL-I: Training load–injury relationship
TL-P: Training load–performance relationship
tRPE: Ratings of technical/cognitive exertion
uRPE: Ratings of upper body exertion
